# Altered Neural Activity during Irony Comprehension in Unaffected First-Degree Relatives of Schizophrenia Patients—An fMRI Study

**DOI:** 10.3389/fpsyg.2017.02309

**Published:** 2018-01-09

**Authors:** Róbert Herold, Eszter Varga, András Hajnal, Edina Hamvas, Hajnalka Berecz, Borbála Tóth, Tamás Tényi

**Affiliations:** Department of Psychiatry and Psychotherapy, Medical School, University of Pécs, Pécs, Hungary

**Keywords:** schizophrenia, irony, theory of mind, social cognition, endophenotype, relatives, functional MRI

## Abstract

Irony is a type of figurative language in which the literal meaning of the expression is the opposite of what the speaker intends to communicate. Even though schizophrenic patients are known as typically impaired in irony comprehension and in the underlying neural functions, to date no one has explored the neural correlates of figurative language comprehension in first-degree relatives of schizophrenic patients. In the present study, we examined the neural correlates of irony understanding in schizophrenic patients and in unaffected first-degree relatives of patients compared to healthy adults with functional MRI. Our aim was to investigate if possible alterations of the neural circuits supporting irony comprehension in first-degree relatives of patients with schizophrenia would fulfill the familiality criterion of an endophenotype. We examined 12 schizophrenic patients, 12 first-degree relatives of schizophrenia patients and 12 healthy controls with functional MRI while they were performing irony and control tasks. Different phases of irony processing were examined, such as context processing and ironic statement comprehension. Patients had significantly more difficulty understanding irony than controls or relatives. Patients also showed markedly different neural activation pattern compared to controls in both stages of irony processing. Although no significant differences were found in the performance of the irony tasks between the control group and the relative group, during the fMRI analysis, the relatives showed stronger brain activity in the left dorsolateral prefrontal cortex during the context processing phase of irony tasks than the control group. However, the controls demonstrated higher activations in the left dorsomedial prefrontal cortex and in the right inferior frontal gyrus during the ironic statement phase of the irony tasks than the relative group. Our results show that despite good task performance, first-degree relatives of schizophrenia patients had alterations in the neural circuits during irony processing. Thus, we suggest that neural alteration of irony comprehension could be a potential endophenotypic marker of schizophrenia.

## Introduction

Human verbal communication is rich in figurative, or nonliteral language (e.g., metaphors, idioms, humor or irony). The comprehension of figurative language requires pragmatic language abilities. Pragmatic ability refers to language usage in context (Levinson, 1983). By using nonliteral constructions, people express their thoughts, feelings, and attitudes to one another in a uniquely emphasized way (Ortony, 1975; Roberts and Kreuz, 1994; Fussell and Moss, 1998). For example, ideas that may be difficult to formulate literally can be communicated effectively using metaphors (Ortony, 1975). Irony, on the other hand, serves various social communicative functions. Based on its linguistic dichotomy or discrepancy it can serve to express politeness, display emotion or humor, or even enhance criticism (Milanowicz, 2013).

Apart from metaphors, irony was found to be one of the most widely used non-literal language construction in social communication, therefore it is supposed to have a special role in human thinking (Kreutz et al., 1996). In an ironic speech act the implicit communicative intent of the speaker is contradictory to what is explicitly expressed. Irony comprehension is a complex process that involves the decoding of the social context as well as the speaker's intention (Sperber and Wilson, 1995, 2002). Integrating the literal expression of the speaker and the social context (Sperber and Wilson, 1995) is indispensable for the interlocutor in order to represent the speaker's mind as well as to recognize that the intention of the speaker is contrary to the literal meaning of the expressed ironic remark. For these reasons, it is necessary for the interpretation of irony to construct a coherent narrative based on contradictory information between the literal meaning of the ironic statement and the context, through the proper interpretation of the speaker's communicative intentions.

There is great interest in investigating pragmatic impairments in various dimensions of figurative language processing in schizophrenia (Herold et al., 2002; Langdon et al., 2002a,b; Tényi et al., 2002; Brüne and Bodenstein, 2005; Thoma and Daum, 2006; Mo et al., 2008; Champagne-Lavau and Stip, 2010; Gavilán and García-Albea, 2011; Colle et al., 2013; Gavilán Ibá-ez and García-Albea Ristol, 2013; Sela et al., 2015; Bosco and Parola, 2017), and there is now extensive evidence for the impairment of irony comprehension itself in schizophrenia (Herold et al., 2002; Sprong et al., 2007; Gavilán and García-Albea, 2011; Rapp et al., 2013; Saban-Bezalel and Mashal, 2017), although not all patients show the deficit (Champagne-Lavau et al., 2012; Varga et al., 2014). Since figurative language has a special role in social communication, it is unsurprising that such deficits can lead to social isolation among patients (Champagne-Lavau and Stip, 2010).

Some authors argue that the ability to attribute beliefs and intentions (i.e., mental states) to others (Theory of Mind, ToM; Premack and Woodruff, 1978; Dennett, 1989) is crucial for irony comprehension (Sperber and Wilson, 1995, 2002). In line with this assumption, several studies found (Winner and Leekam, 1991; Happé, 1993; Sullivan et al., 1995; Winner et al., 1998) that second-order ToM is necessary for irony interpretation. However, other studies did not find any connection between the two (Martin and McDonald, 2005; Ziv et al., 2011). In addition, Bosco and Gabbatore (2017) found that ToM abilities could only partially explain the performance of typically developing children in tasks measuring the comprehension of deceitful and ironic communicative acts. Moreover, studies about pragmatic disability in individuals with traumatic brain injuries pointed out that the interpretation of irony requires both ToM skills as well as executive functions (Martin and McDonald, 2005; McDonald et al., 2014; Bosco et al., 2017a). Difficulties in figurative language comprehension in schizophrenia have also been related to a variety of factors. Several studies found a relationship between irony processing and ToM in schizophrenia (Langdon et al., 2002a,b; Gavilán and García-Albea, 2011), however some of them did not (Mo et al., 2008). Furthermore, results showed that defective irony comprehension was influenced by executive functions (Herold et al., 2004; Champagne-Lavau and Stip, 2010; Varga et al., 2014) and higher-level language skills among patients (Colston and Katz, 2005; Rapp et al., 2010, 2012, 2014). In addition, a model of Leitman et al. (2006) proposed that deficits in prosody perception would be a cause of sarcasm detection impairment in schizophrenia. Taking all these into consideration, it can be stated that the exact relation between irony and ToM is still not completely clear and it is important to point out that these two elements interact but they do not correspond.

Previous studies showed that pragmatic incompetence may precede the onset of schizophrenia (Dodell-Feder et al., 2014; Sullivan et al., 2016), while Bambini et al. (2016) pointed out that pragmatic impairment is diffuse and independent from clinical symptoms, and it can be linked to the underlying biology of schizophrenia, considering it a core feature of the disease. This in turn suggests that pragmatic deficits may have an endophenotypic nature. It has been proposed that the genetic risk for clinical illnesses is mediated by endophenotypes, thus the study of endophenotypes is a promising field of schizophrenia research (Gottesman and Gould, 2003), intending to identify intermediate phenotypes, which are close to the biological essence of the disease, and which would fill the gap between the genes responsible for the development of the disease, and the clinical symptoms (Hajnal et al., 2014, 2016).

The growing interest in identifying endphenotypes has led to the increase in the number of functional imaging studies of first-degree relatives of schizophrenic individuals, as they carry some of the risk genes of the disease, while they are unaffected by the biological changes and side-effects caused by acute psychotic states or pharmaceutical treatment (MacDonald et al., 2009).

Recently, an increasing number of fMRI studies have investigated the neural basis of irony comprehension. Since irony is a special figure of thought, depending upon different cognitive operations, many studies found activations in some typical regions of the ToM network (Uchiyama et al., 2006; Wang et al., 2006b; Shamay-Tsoory and Aharon-Peretz, 2007; Wakusawa et al., 2007; Rapp et al., 2010, 2012; Shibata et al., 2010; Bohrn et al., 2012; Spotorno et al., 2012; Bosco et al., 2017b), as well as of the semantic and executive system (Eviatar and Just, 2006; Rapp et al., 2010, 2012; Spotorno et al., 2012; Bosco et al., 2017b). In line with previous suggestions (Spotorno et al., 2012; Obert et al., 2016) Van Ackeren et al. (2016) demonstrated using connectivity analysis that ToM and language networks interact while interpreting indirect speech acts.

As far as we know, to date no one has explored the neural correlates of figurative language comprehension in first-degree relatives of schizophrenic individuals. Furthermore, there are only two fMRI studies about the neural correlates of the comprehension of ironic remarks in schizophrenia (Rapp et al., 2013; Varga et al., 2013). Because of this, in the present study our aim was to examine the neural correlates of irony understanding in schizophrenic patients, in their first-degree relatives and in a matched control group, with the same methods as we used in our previous study (Varga et al., 2013).

In Rapp et al. (2013) 15 female schizophrenic patients were examined. They made significantly more mistakes in the irony tasks on a behavioral level than the controls, and showed decreased activations during irony comprehension in the posterior medial prefrontal cortex, the left insula, the right middle temporal gyrus, and bilateral postcentral gyrus, while exhibiting increased activations in the left parahippocampal gyrus. Their main study hypothesis was that there is a dysfunction of the frontotemporal language system in schizophrenia. In line with their predictions they found decreased activation in the right middle temporal gyrus in the schizophrenic group.

In Varga et al. (2013) irony comprehension and the correlating brain activations were analyzed in schizophrenic patients. In Varga et al. (2013) we separately examined the processing of the context phase as well as the ironic statement phase of the irony tasks. The context phase consisted of a description of a social situation with two interlocutors, while the ironic statement phase consisted of one of the interlocutors' ironic remark. We found that in the irony comprehension tasks healthy controls performed significantly better than schizophrenic patients. In addition, the sequential analysis of fMRI data showed that the two groups manifested significantly different brain activation patterns both in the context phase and in the statement phase. While patients exhibited stronger activations in the parietal and frontal areas in the context phase of irony tasks, the healthy controls showed higher activations in frontal, temporal and parietal regions during the ironic statement phase of the irony task.

To address, whether altered comprehension of irony is a trait-like marker of liability to schizophrenia or, alternatively, a biomarker of the illness itself, we tested for its presence in schizophrenia patients' first-degree relatives. It was hypothesized that unaffected first-degree relatives of schizophrenic patients would present irony comprehension impairment reflecting an intermediate phenotype compared to patients and healthy controls. We also assumed that the first-degree relatives would exhibit a different brain activation pattern compared to controls and patients with schizophrenia.

## Materials and methods

### Participants

We included 12 (6 males and 6 females, from 22 to 51 years old) patients with schizophrenia (schizophrenia group, SG), 12 (6 males and 6 females, from 26 to 53 years old) first-degree relatives of schizophrenia patients (relative group, RG) and 12 (5 males and 7 females, from 26 to 55 years old) healthy adults (control group, CG), in the study.

The SG consisted of patients diagnosed with paranoid schizophrenia, and met the diagnostic criteria of DSM-IV. We recruited patients from the psychosis unit of the Department of Psychiatry and Psychotherapy, University of Pécs. They were all in remission, as specified by the remission criteria of schizophrenia (Andreasen et al., 2005), more specifically the key items of Positive and Negative Syndrome Scale (PANSS) (delusions, unusual thought content, hallucinatory behavior, conceptual disorganization, mannerism/posturing, blunted affect, social withdrawal, lack of spontaneity) had been mild or less (≤3) for at least 6 months prior to taking part in the study. In order to confirm their diagnosis, we rated participants using the Schedule for Affective and Schizophrenic Disorders–Lifetime Version (Endicott and Spitzer, 1978). We excluded all patients who had a history of substance abuse, neurological disorder, and mental retardation or cognitive deficits unrelated to schizophrenia. Patients were on maintenance treatment with antipsychotic pharmaceuticals.

Contact to the relatives was established through schizophrenic patients from the psychosis unit of the Department of Psychiatry and Psychotherapy, University of Pécs. Schizophrenic probands fulfilled the diagnostic criteria of DSM-IV, they were rated using the Schedule for Affective and Schizophrenic Disorders–Lifetime Version to confirm their diagnoses (Endicott and Spitzer, 1978), and they had no history of substance abuse, neurological disorder, or mental retardation. The group of first degree relatives was composed of 10 parents, and 2 brothers. A physical examination was carried out for each participant of the RG prior to the experiment, and showed no abnormalities. None of them have a previous history of traumatic brain injury, mental illness or other brain diseases. They were also given the Hungarian version of Structured Clinical Interview for DSM-IV (SCID) (First et al., 2007) to exclude subjects with psychiatric illnesses. Relatives had no lifetime evidence of psychotic disorder, and no lifetime exposure to antipsychotic medication.

For the CG, we recruited participants through newspaper advertisements and the local unemployment office. None of them had a history of psychiatric illnesses, personally or within their families. The presence of neurological morbidity, dependence on psychoactive substances (excluding caffeine and tobacco) were also ruled out. Controls were screened with SCID. The age, the gender and the IQ scores of the SG and the RG were matched to the characteristics of the CG, as there were no significant differences in gender, IQ (*p* = 0.136, n.s.) and age (*p* = 0.195, n.s.) between the three groups (Table 1).

**Table 1:** Demographic and clinical data and task performances in the CG, the SG, and the RG.

**Variable**	**CG (*****n*** = **12)**		**SG (*****n*** = **12)**		**RG (*****n*** = **12)**		***p*-value^e^**
	**Mean**	***SD***	**Mean**	***SD***	**Mean**	***SD***	
Gender (male/female)	5/7		6/6		6/6		
Age (years)	37.00	9.08	36.88	7.99	42.9	10.52	0.195^a^
Full Scale IQ^c^	118	10.47	107.76	12.38	116.8	7.13	0.136^a^
PANSS (total)	65.70	13.45					
PANSS (positive)	14	4					
PANSS (negative)	18.02	5.47					
PANSS (depression)	9.38	3.39					
PANSS (general)	34.15	7.30					
Age at onset (years)^f^	27.69	6.77					
Duration of illness (years)	10	6.74					
Response accuracy in tasks during scanning^d^							
Irony tasks	0.50	0.50	1.46	1.19	0.6	1.07	0.033^b^
Control tasks	0.16	0.38	0.84	1.46	0.4	0.5	0.303^b^

a *Mann-Whitney U-test*.

b *Kruskall-Wallis one-way analysis of variance (ANOVA) by ranks*.

c *General IQ*.

d *The mean and SD of the number of incorrect answers*.

e *Statistically significant differences, two-tailed p < 0.05, uncorrected*.

f *Age of onset was defined as the presentation of psychotic symptoms in the context of functional decline*.

We measured the general intelligence of participants using the Hungarian version of Wechsler Adult Intelligence Scale (WAIS; Wechsler, 2007). All participants were right-handed, estimated by Edinburgh handedness inventory (Oldfield, 1971). After the participants were given a comprehensive description of the study, written informed consents were obtained. We conducted our investigation adhering to institutional guidelines. Ethical perspectives were established in compliance with the latest version of the Declaration of Helsinki. The Committee on Medical Ethics of University of Pécs accepted the proposal for this study (the number of the ethical permit is 6416).

### Stimuli

For the present experiment, we used a part of a test battery (with the exception of the irony with linguistic help tasks) previously created for the fMRI investigation of irony comprehension in Varga et al. (2013).

Two experimental conditions were used: irony (I) and control (C) conditions. In the I condition 15 short scenarios about social situations containing ironic remarks were presented as irony tasks, and in the C condition 15 scenarios based on physical causality were presented as control tasks. Each task was comprised of three different phases, as each of them started with a context phase followed by a statement phase and a question/answer phase. The question/answer phase was created to test whether the statement was correctly understood. The appropriate response was “Yes” if the comment was true, and “no” if the comment was false.

The irony tasks started with a context phase describing the background circumstances of the social situation with two interlocutors. The context phase described the perspective, the implicit emotional state and the communicative intent of the characters. In order to be able to choose between the competing interpretations of the ironic statement, participants had to take into consideration the content of the context phase. The statement phase consisted of an ironic remark/statement of one of the interlocutors, in which the literal meaning was the opposite of the intended one. The control tasks contained simple physical causalities, which entailed the representation of non-intentional causal links.

The following scenario is an example for the irony tasks:

“*Context phase: Joe went home from school and told his father that he had failed his maths test. His father said:*

Ironic statement phase: Oh boy, you just made my day!

*Question-answer phase: Did Joe's father think that Joe made his day?”* (Varga et al., 2013, pp. 240.)

The following scenario is an example for the control tasks:

“*Context phase: It is raining all day. There is so much water flowing down the water-spout that it floods the whole yard*.

*Statement phase: The huge amount of water renders the entire yard heavily muddy*.

*Question-answer phase: Does the yard stay dry after the day-long rain?”* (Varga et al., 2013, pp. 240.)

We used auditory stimuli, as opposed to reading, in order to decrease individual differences in stimuli processing. Scenarios were matched in syntactic structure and semantic complexity. We matched the length of the scenarios across the different conditions (I, C) as well as the length of the different phases (context, statement and question/answer phase) of the scenarios. We found no significant difference between them. The average length was 14.62 s (1.01 SD). It was 14.85 s (1.07 SD) for the I and 14.38 s (1.03 SD) for the C. The average duration of the context phase was 8.5 s (1.01 SD). It was 8.9 s (1.78 SD) in the I and 7.43 s (0.64 SD) in the C. The average duration of the statement phase was 3.24 s (0.68 SD). It was 2.97 s (0.3 SD) in the I and 3.99 s (0.6 SD) in the C. The average length of the question/answer phase was 2.85 s (0.45 SD). It was 2.9 s (0.43 SD) in the I and 2.96 s (0.5 SD) in the C.

Between trials participants had 5–7 s to answer the questions. All participants were capable of responding within the given time, otherwise their data would have been excluded from the analysis.

Participants were tested with examples of scenarios before fMRI scanning. The example scenarios were similar to those used during scanning. All participants demonstrated their understanding of task requirements by successfully completing a series of practice tasks as well as showing appropriate use of task controls. Two of the authors (VE, HA) also conducted verbal exploration of the participants to check if they understood the tasks appropriately.

### Activation paradigm

As for the activation paradigm, we also refer to our previous study (Varga et al., 2013). Each task had a specific structure, starting with a context phase (1), followed by a 2–4-s-long (jittered) inter-stimulus interval. Next the statement phase (2) appeared which was followed by a comprehension question (3). Individual tasks were followed by inter-trial intervals of 5–7 s (jittered). Following the question, participants were required to give yes/no answers by pressing a button either with their thumb (meaning yes) or their index finger (meaning no), as quickly as possible. The experimental protocol consisted of 30 tasks and was conducted in one sitting; scanning time of the entire session lasted approximately 20 min. During the fMRI data analysis, we examined each phase of the tasks as a separate event, so each session amounted to a total of 90 (=30 × 3) events. Scenarios were presented in random order, as we tried to model real life, where ironic remarks cannot be anticipated. The order in which the tasks appeared was the same for each group. Stimuli were presented with NordicNeuroLab fMRI Hardware (VisualSystem, AudioSystem, ResponseGrip, SyncBox). During scanning, participants' answers were recorded and saved. The accuracy of the responses was assessed subsequently. Correct answers were regarded as scores, representing participants' performance in the irony (I score) and the control tasks (C score).

### Functional MRI data acquisition

Functional magnetic resonance (MR) imaging was performed on a 3T MR scanner (Siemens Magnetom Trio, Siemens AG, Erlangen, Germany) with 12-channel phased array TIM head coil for radio frequency reception. We used a standard EPI sequence to obtain functional MR images with the following parameters: TR (repetition time): 2,000 ms; TE (echo time): 36 ms; voxel size: 2 × 2 × 3 mm, field of view: 192 × 192 mm; 23 axial slices with a thickness of 4-mm (no gap), interleaved slice order to avoid crosstalk; 76° flip angle; 1,360-Hz receiver bandwidth. We acquired 567 volumes per session. Anatomical images were acquired using a magnetization prepared rapid gradient echo (MP-RAGE) sequence (TR: 1,900 ms; TE: 3.44 ms, 9° flip angle, 180-Hz receiver bandwidth, 0.9 × 0.9 × 0.9 mm^3^ isotropic voxel size).

### Demographic, clinical, and behavioral data analysis

We used Statistical Package for the Social Sciences (spss; SPSS Inc., Chicago, IL, USA; Nie, 1975) version 20 for Windows to do the statistical analysis of experimental task performance, full scale IQ and demographic data. We checked data distribution with Kolmogorov–Smirnov goodness of fit. As distributions did not prove to be normal, Kruskal-Wallis one-way analysis of variance (ANOVA) by ranks and Mann-Whitney *U*-test were performed to compare group medians across the experimental conditions, age and IQ.

### Functional MRI data analysis

To analyse functional data sets we used FSL 5.0.9. (FMRIB's Software Library, www.fmrib.ox.ac.uk/fsl). We used FEAT (FMRI Expert Analysis Tool) Version 6.00, part of FSL for FMRI data processing. We used the following pre-statistics processing; MCFLIRT for motion correction (Jenkinson and Smith, 2001); BET for non-brain removal (Smith, 2002); spatial smoothing using a Gaussian kernel of FWHM 5 mm; grand-mean intensity normalization of the entire 4D dataset by a single multiplicative factor; highpass temporal filtering (Gaussian-weighted least-squares straight line fitting, with sigma = 25.0 s).

Following preprocessing time-series statistical analysis was done using FILM (FMRIB's Improved Linear Model) with local autocorrelation correction (Woolrich et al., 2001). For modeling blood oxygenation level-dependent (BOLD) changes in each phases of the scenarios, separate regressors were identified for the representation of the context phase, the statement phase, as well as the question-answer phase in both I and C conditions. Contrasts of regressors were further specified: context phase: I>C, statement phase: I>C question-answer phase: I>C. I>C contrast of regressors were defined so that the confounding factor of basic semantic processing would be eliminated.

To test for variations in activation patterns within as well as between the three group the resulting first-level contrast images were entered into higher-level analyses.

FLIRT (Jenkinson and Smith, 2001; Jenkinson et al., 2002) was used for the registration to high resolution structural and standard space images. By using FNIRT nonlinear registration (Andersson et al., 2007a,b), registration from high resolution structural to standard space was further refined.

We used FLAME (FMRIB's Local Analysis of Mixed Effects) stage 1 and stage 2 to conduct higher-level analysis (Beckmann et al., 2003; Woolrich et al., 2004; Woolrich, 2008). Z (Gaussianised T/F) statistic images were thresholded using clusters determined by Z > 2.3 and a (corrected) cluster significance threshold of *P* = 0.05 (Worsley, 2001). For display purposes, images were rendered on a mean anatomical brain volume of all subjects in standard space.

## Results

### Behavioral results

Demographic data, WAIS scores and response accuracy in the tasks are summarized in Table 1. In the irony condition, significant differences were found between the performance of the three groups (*p* = 0.033). The results of further analysis (Mann-Whitney *U*-test) revealed no significant between group difference between the CG and the RG (*p* = 0.522, n.s.), however between group differences were significant between the CG and the SG (*p* = 0.020) as well as between the RG and the SG (*p* = 0.030). As for the performance of the control condition, no significant differences were found between the three groups (*p* = 0.303 n.s.; Table 1).

### Functional MRI results

#### Significant brain activations during the context phase (irony vs. control task contrasts) of the tasks

*Within-group activations*:

The *CG* had significant activations in the precuneus in the left hemisphere and in the temporo-parietal junction (TPJ) in the left as well as in the right hemisphere in the I>C contrast.

The *SG* recruited the inferior parietal lobule (IPL) in the left hemisphere, which also reaches the TPJ and the insula in the left hemisphere. They also activated the insula in the right hemisphere, the middle temporal gyrus (MTG) in the right hemisphere, the IPL in the right hemisphere, the dorsolateral prefrontal cortex DLPFC in the right hemisphere, the posterior cingulum in the left hemisphere and the thalamus in the left hemisphere in the I>C contrast.

In the same contrast, the *RG* recruited the precuneus in the left hemisphere, a cluster which also reaches the superior temporal gyrus (STG), the TPJ, MTG and the posterior cingulum in the left hemisphere. They also recruited the DLPFC in the left hemisphere and the paracingulate gyrus in the right hemisphere in the I>C contrast. Results of the within-group activations during the context phase (I>C contrast) are summarized in Table 2.

**Table 2:** Significant activations in control subjects, in schizophrenia patients and in relatives of schizophrenia patients during the context phase of the irony tasks.

**Region**	**Control group**						**Schizophrenic group**						**Relative group**					
	**Hem**	***x***	***y***	***z***	**Zmax**	**Voxel**	**Hem**	***x***	***y***	***z***	**Zmax**	**Voxel**	**Hem**	***x***	***y***	***z***	**Zmax**	**Voxel**
**CONTEXT PHASE (WITHIN-GROUP ACTIVATIONS)**																		
SFG/DLPFC							R	36	40	38	3.88	734	L	−38	42	34	4.1	1,558
Paracingulate Gyrus													R	4	14	44	4.21	1,083
MTG posterior division							R	50	−36	0	5.06	888						
Posterior Cingulum							L	−4	−16	42	5.57	359						
IPL							R	60	−14	26	3.63	827						
IPL							L	−38	−34	40	5.17	11,829						
TPJ							L	−42	−54	18	4.82							
Insula							L	−32	26	2	4.81							
Precuneus	L	−2	−60	40	3.97	1,112							L	−12	−52	34	5.7	12,639
STG posterior division													L	−48	−42	6	5.15	
TPJ													L	−58	−50	16	5.14	
MTG posterior division													L	−60	−30	−4	5.13	
Posterior Cingulum													L	−4	−50	32	4.89	
TPJ	L	−58	−52	24	4.75	972												
TPJ	R	44	−48	18	4.09	368												
Insula							R	34	20	12	3.89	931						
Thalamus							L	−8	−14	−4	3.51	333						
							**Schizophrenic group** > **Control group**						**Relative group** > **Control group**					
**CONTEXT PHASE (BETWEEN-GROUP ACTIVATIONS)**																		
SFG/DLPFC							R	40	46	22	3.73	714	L	−42	40	30	3.59	469
IFG (pars triangularis)							L	−50	40	2	4.42	456						
IFG (pars opercularis)							L	−42	12	24	4.15	1,320						
MFG/DLFC							R	52	8	38	3.43	520						
IPL							R	60	−14	26	3.56	528						
IPL							L	−44	−46	46	4.21	1,132						

*During the context phase, we found significant between-group activations only in the SG>CG and in the RG>CG*.

*x, y, z coordinates are in millimeters, Montreal Neurological Institute (MNI) system*.

*Selected local maxima are shown. Z (Gaussianised T/F) statistic images were threshold using clusters determined by Z > 2.3 and a (corrected) cluster significance threshold of P = 0.05. In case of the larger clusters (5,000–13,000 voxels) we listed the output of the cluster breakdown as well*.

*BA, Brodmann area; Hem, hemisphere; Voxel, number of voxels; L, left; R, right; SFG, Superior Frontal Gyrus; DLPFC, Dorsolateral Prefrontal Cortex; MFG, Middle Frontal Gyrus; DLFC, Dorsolateral Frontal Cortex; IFG, Inferior Frontal Gyrus; STG, Superior Temporal Gyrus; MTG, Middle Temporal Gyrus; TPJ, Temporo-parietal Junction; IPL, Inferior Parietal Lobule*.

Between-group comparisons:

Between group comparison of the I>C contrast revealed significantly stronger activations of the pars opercularis of the left inferior frontal gyrus (IFG), the left IPL, the right DLPFC, the right IPL, the right middle frontal gyrus (MFG), and the pars triangularis of the IFG in the SG compared to the CG.

Significantly stronger activation of the left DLPFC was found in the RG compared to the CG in the I>C contrast.

**Figure 1:**
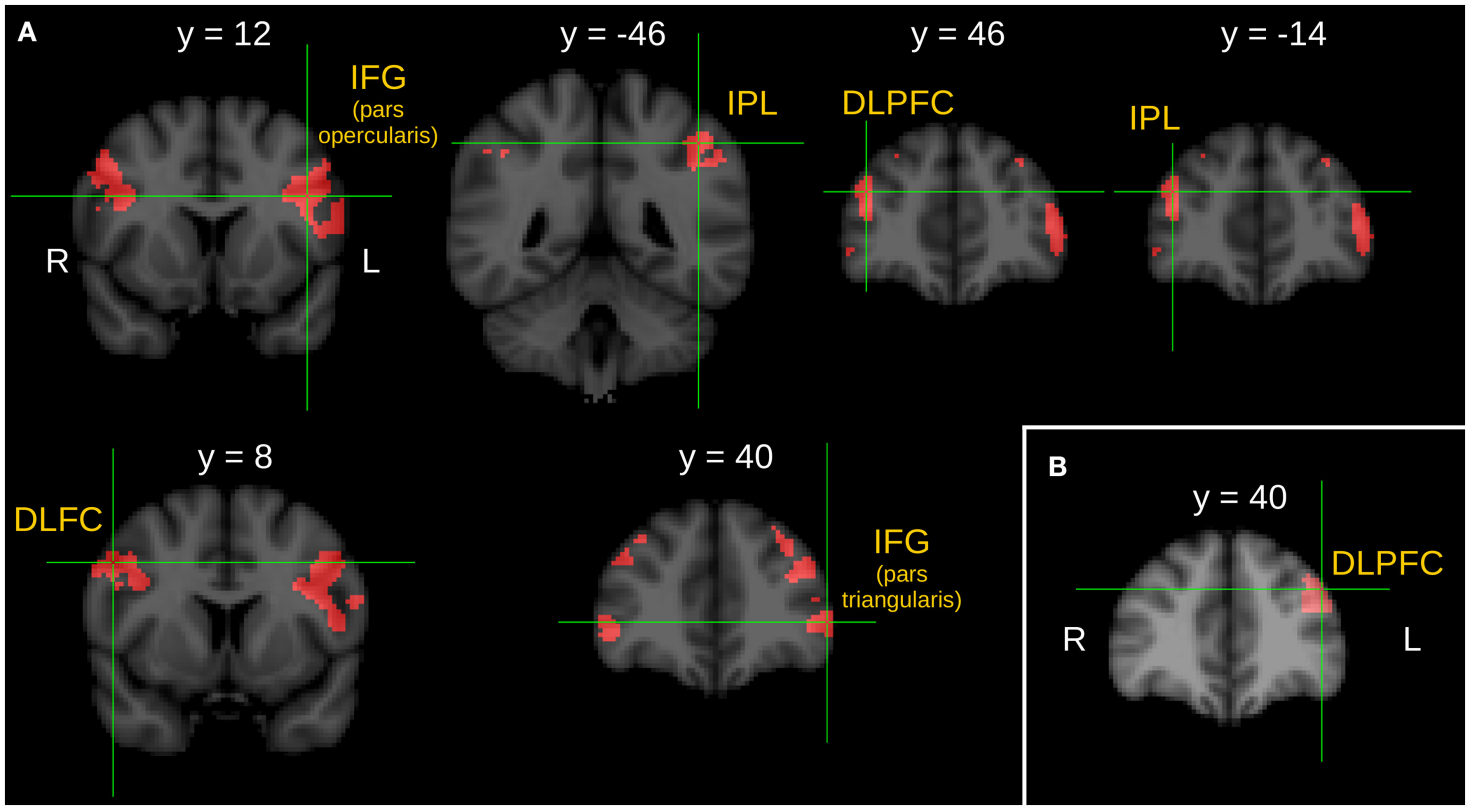
Between group comparison of the *I* > *C* contrast during the context phase. **(A)** Brain areas show significantly greater activity in schizophrenic patients than in healthy control subjects. **(B)** Brain area shows significantly greater activity in first-degree relatives of schizophrenia patients than in healthy control subjects. *Z* statistic images were threshold using clusters determined by *Z* > 2.3 and a corrected cluster significance threshold of *P* = 0.05. *L*, left; *R*, right; IFG, inferior frontal gyrus; IPL, inferior parietal lobule; DLPFC, dorsolateral prefrontal cortex; DLFC, dorsolateral frontal cortex.

Note that we did not find any stronger activation in the CG compared to the SG, in the CG compared to the RG, in the SG compared to the RG nor in the RG compared to the SG during the between-group comparison of the I>C contrasts. Results of the between-group comparisons during the context phase (I>C contrast) are illustrated in Figure 1.

#### Significant brain activations during the statement phase (irony vs. control task contrasts) of the tasks

Within-group activations:

In the I>C contrast, the CG was found to engage the left temporal pole (TP) (reaching the left STS), the right superior temporal sulcus (STS) (reaching the right TP), the left dorsomedial prefrontal cortex (DMPFC) extending into the right DMPFC and the left posterior cingulum, a cluster which also reaches the right posterior cingulum.

The *SG* engaged the IPL in the left hemisphere as well as the pars orbitalis of the IFG in the left hemisphere in the I>C contrast.

The *RG* activated the left STG, the right posterior STS and the left IFG pars triangularis in the I>C contrast. Results of the within-group activations during the statement phase (I>C contrast) are summarized in Table 3.

**Table 3:** Significant activations in control subjects, in schizophrenia patients and in relatives of schizophrenia patients during the ironic statement phase of the irony tasks.

**Region**	**Control group**						**Schizophrenic group**						**Relative group**					
	**Hem**	***x***	***y***	***z***	**Zmax**	**Voxel**	**Hem**	***x***	***y***	***z***	**Zmax**	**Voxel**	**Hem**	***x***	***y***	***z***	**Zmax**	**Voxel**
**IRONIC STATEMENT PHASE (WITHIN-GROUP ACTIVATIONS)**																		
SFG/DMPFC	L	−4	52	38	5.03	1,961												
IFG (pars triangularis)							L	−48	32	−4	3.66	266	L	−52	22	14	4.21	776
STG posterior division													L	−64	−34	2	4.56	1,234
STS	R	56	−14	−8	6.14	7,111							R	48	−36	6	5.59	1,130
TP	R	66	−16	0	5.5													
TP	L	−54	2	−14	6.34	9,085												
STS anterior division	L	−58	−8	−8	6.12													
STS posterior division	L	−54	−40	0	6.04													
Posterior Cingulum	L	−10	−44	36	4.61	1,782												
IPL							L	−52	−56	36	3.69	830						
							**Control group**>**Schizophrenic group**						**Control group**>**Relative group**					
**IRONIC STATEMENT PHASE (BETWEEN-GROUP ACTIVATIONS)**																		
SFG/DMPFC													L	−4	50	38	3.95	358
MFG/DLPFC							R	44	36	22	4.37	1,769						
IFG (pars triangularis)													R	58	28	6	3.74	455
STG anterior division							L	−64	−14	−2	3.49	322						
IPL							R	52	−42	30	3.34	435						
IPL (Supramarginal Gyrus)							L	−58	−28	28	3.32	298						

*During the ironic statement phase, we found significant between-group activations only in the CG > SG and in the CG > RG*.

*x, y, z coordinates are in millimeters, Montreal Neurological Institute (MNI) system*.

*Selected local maxima are shown. Z (Gaussianised T/F) statistic images were threshold using clusters determined by Z > 2.3 and a (corrected) cluster significance threshold of P = 0.05. In case of the larger clusters (5,000–10,000 voxels) we listed the output of the cluster breakdown as well*.

*BA, Brodmann area; Hem, hemisphere; Voxel, number of voxels; L, left; R, right; SFG, Superior Frontal Gyrus; DLPFC, Dorsolateral Prefrontal Cortex; MFG, Middle Frontal Gyrus; DMPFC, Dorsomedial Prefrontal Cortex; IFG, Inferior Frontal Gyrus; STG, Superior Temporal Gyrus; STS, Sulcus Temporalis Superior; TP, Temporal Pole; IPL, Inferior Parietal Lobule*.

Between-group comparisons:

Between-group comparison of the I>C contrasts during the statement phase detected significantly stronger activations in the right DLPFC, in the right IPL, in the anterior division of the left STG and in the left IPL in the CG compared to the SG.

Significantly stronger activations of the part triangularis of the right IFG and left DMPFC were found in the CG compared to the RG in the I>C contrast.

**Figure 2:**
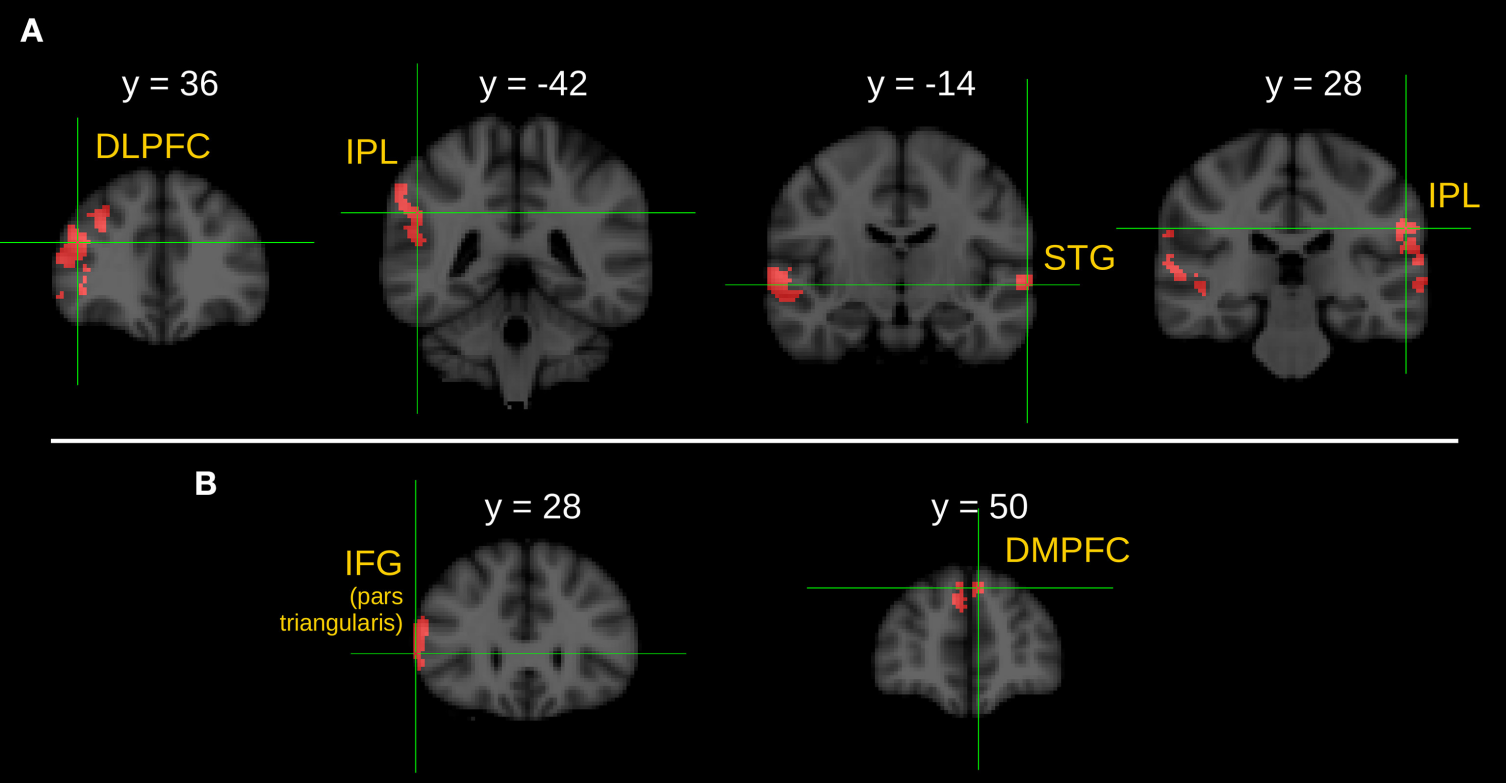
Between group comparison of the *I* > *C* contrast during the ironic statement phase. **(A)** Brain areas show significantly greater activity in healthy control subjects than in schizophrenic patients. **(B)** Brain areas show significantly greater activity in healthy control subjects than in first-degree relatives of schizophrenia patients. *Z* statistic images were threshold using clusters determined by *Z* > 2.3 and a corrected cluster significance threshold of *P* = 0.05. *L*, left; *R*, right; DLPFC, dorsolateral prefrontal cortex; IPL, inferior parietal lobule; STG, superior temporal gyrus; IFG, inferior frontal gyrus; DMPFC, dorsomedial prefrontal cortex.

Note that we did not find any stronger activation in the SG compared to the CG, in the RG compared to the CG, in the SG compared to the RG nor in the RG compared to the SG during the between-group comparison of the I>C contrasts. Results of the between-group comparisons during the statement phase (I>C contrast) are illustrated in Figure 2.

#### Activation patterns during the question-answer phase (irony vs. control task contrasts) of the tasks

No significant within-group or between-group differences were observed during the question-answer phase of the tasks.

## Discussion

The current study investigated irony comprehension and its neural correlates in schizophrenic patients (SG) and in unaffected first-degree relatives of patients (RG) compared to healthy adults (CG). In keeping with previous studies (Rapp et al., 2013; Varga et al., 2013), the performance of the CG was significantly better during irony comprehension tasks than that of the SG, as well as significant between group differences were observed during the sequential analysis of fMRI data.

The performance of the unaffected first-degree relatives of schizophrenic patients showed no significant differences compared to the CG during irony comprehension. However, the sequential analysis of fMRI data of the two groups revealed significantly different brain activation patterns.

Although there was a significant difference in the performance of the irony tasks between the RG and the SG, the two groups did not differ significantly in their brain activation patterns, neither in the context phase nor in the ironic statement phase.

### Within-group activations during the context phase (irony vs. control task contrasts)

Similarly to the results in our previous study (Varga et al., 2013), while processing the context phase, the activation of the left precuneus and the left and right TPJ was observed in the *CG*. In a previous meta-analysis (Bohrn et al., 2012), irony processing was correlated with activations in the midline structures, including the precuneus, furthermore, the precuneus has been repeatedly found to be associated with self-reflection, autobiographical memory, and mental imagery (Cavanna and Trimble, 2006; Schurz et al., 2014). In Schnell et al. (2016) the precuneus was assumed to be responsible for inferential meaning construction as well as for pragmatic processing.

The TPJ was also found to be active in irony comprehension (Bosco et al., 2017b). More specifically, the left TPJ has an important role in understanding communicative intentions (Walter et al., 2004; Saxe and Wexler, 2005; Ciaramidaro et al., 2007; Bosco et al., 2017b). Increasing evidence from neurocognitive studies indicates that the right TPJ also plays a crucial role in several aspects of social cognition (Decety and Lamm, 2007; Schurz et al., 2014). All these are important capacities for the comprehension of the social context of a pragmatic phenomenon.

Remarkably, while processing the social context phase, the *SG* mobilized widespread brain areas not only in the temporo-parietal regions, but also in the frontal, as well as in the subcortical regions, which suggest a great effort to cope with substantial linguistic, cognitive, and emotional load during the comprehension of the social context of irony (Varga et al., 2013; Bosco et al., 2017b).

Similarly to the SG, while comprehending the context phase, the *RG* also recruited widespread areas in prefrontal, frontal, temporal or parietal regions with local maximums in the left precuneus, in the left DLPFC and in the right paracingulate gyrus. As we have mentioned earlier, the precuneus has been identified in irony processing (Bohrn et al., 2012), and is assumed to have a major role in pragmatic processing (Schnell et al., 2016).

The left DLPFC has been associated with different cognitive processes, such as working memory and episodic memory retrieval (Gilbert et al., 2006), while recently, high executive demand during irony processing has also linked to the activation of the left DLPFC (Spotorno et al., 2012; Bosco et al., 2017b). An important finding of this present study is that in the context phase the RG activated the left DLPFC significantly stronger than the CG, which we will discuss in more details in the following paragraphs. Interestingly, context processing, which is an executive ability that depends on several cognitive processes, is believed to be subserved by the DLPFC (Reilly et al., 2017).

In previous studies, activations in the paracingulate cortex were associated with performance on tasks engaging executive cognitive processes (Fornito et al., 2004) and its role was also found in language processing (Herholz et al., 1996; Crosson et al., 1999). Moreover, activations in the paracingulate gyrus were associated with tasks requiring ToM skills (Brunet-Gouet and Decety, 2006), and this region was found to play a role in predicting future intentional social interactions (Walter et al., 2004) as well. It can be proposed that the increased activation of the paracingulate gyrus observed in the RG suggests a mentalizing effort to understand the social context of a pragmatic situation.

### Between-group activations during the context phase (irony vs. control task contrasts)

In the between-group comparison, the SG showed markedly stronger activations in several brain areas compared to the CG. In previous studies, higher activation of the left IFG (BA44) has been connected with schizotypal personality traits, high risk for psychosis, as well as established schizophrenia in various paradigms including irony tasks (Kircher et al., 2007; Rapp et al., 2010; Sabb et al., 2010). More specifically, BOLD responses in the left pars triangularis of the IFG (BA 45) have been classically detected during semantic processing of speech (Özyürek, 2014). In addition, in previous studies this region has been considered to play a role in semantic processing of irony (Rapp et al., 2012; Obert et al., 2016; Bosco et al., 2017b).

Additionally, the recruited pars opercularis of the left IFG and the left IPL/aIPS are considered to be parts of the human “mirror neuron system” (MNS) (Rizzolatti and Craighero, 2004; Molenberghs et al., 2012). Repeated findings suggest the involvement of the MNS in nonliteral language processing (McGeoch et al., 2007; Rapp et al., 2010, 2012), thus we can speculate that the activations of these MNS regions may reflect an intrinsic automatic attempt of patients with schizophrenia to simulate complex social contexts based on their previous social experiences (Gallese, 2007). Interestingly, same authors assume that deficits in the MNS in schizophrenia (Mehta et al., 2012) might be responsible for the impaired ability to remove ambiguity from communicative signals (Salvatore et al., 2012).

The right IPL also showed significantly higher activation in the SG compared to the CG. The right IPL was activated during self-reference (Abu-Akel and Shamay-Tsoory, 2011) and self-other discrimination (Uddin et al., 2006), while simulating a complex social context based on the individual's previous social experience, which is consistent with the above described MNS process. In addition, based on their PET study, Ruby and Decety (2001) suggest that the right IPL is specifically involved in differentiating between self-produced actions as opposed to actions generated by others.

In the present experiment, higher activations in the right anterior DLPFC and in the right MFG were also detected in the SG compared to the CG. The right DLPFC reflects the engagement of a control mechanism for top-down biasing of context processing in resource-demanding memory tasks (Kompus et al., 2009). The activation of this region has been associated with decision making in risky social situations (Rodrigo et al., 2014), with encoding and/or updating context information (D'Ardenne et al., 2012) and with anticipation or attention to emotional judgment (Grimm et al., 2008). Moreover, context processing difficulties were found to be associated with right lateralized MFG dysfunction in schizophrenia (Poppe et al., 2015).

Unlike previous works, we analyzed the context phase separately from the statement phase. In our opinion, in the current study, the presented stronger activations in the SG compared to the CG during understanding the social context phase can be explained as compensatory activations for cognitive and language deficits, together with an extra effort for the offline simulation and the embodied understanding of the characters' intentions. All these correspond to schizophrenic patients' difficulties in context processing (Hemsley, 2005), and could substantially account for their difficulties in social cognition (Green et al., 2005).

While evaluating the between-group differences, we found that the RG showed significantly increased activations in the left DLPFC compared to the CG. Increased activations in left DLPFC were related to inhibitory attentional control while processing ambiguous semantic meaning (Hoenig and Scheef, 2009). Furthermore, the left DLPFC was also recruited during irony comprehension (Spotorno et al., 2012; Bosco et al., 2017b). Spotorno et al. (2012) suggested that significant activation in the left DLPFC shows high executive load that is necessary to comprehend the complex forms of language, like irony. In our opinion, in the present research this higher activation of the RG in the left DLPFC during processing contextual information may be related to their extra cognitive effort in context processing and may be interpreted as a compensatory activity. Since the RG performed the irony tasks at the CG level, the found compensatory activation appears to be working to a satisfactory extent to help understand social context.

### Within-group activations during the ironic statement phase (irony vs. control task contrasts)

In line with previous neuroimaging studies (e.g., Uchiyama et al., 2006; Wang et al., 2006a,b; Wakusawa et al., 2007; Shibata et al., 2010; Rapp et al., 2012; Spotorno et al., 2012; Bosco et al., 2017b), we found that the *CG* recruited extended cerebral networks during the comprehension of irony. As we investigated the neural correlates of the ironic statement phase, widespread activations were registered in several temporal and frontal regions in the CG.

We found a left temporal lobe activation with a local maximum in the left TP, a cluster which reaches the posterior STS (Uchiyama et al., 2006; Shibata et al., 2010), as well as activations in the right STS (a cluster which reaches the right TP), in the bilateral DMPFC (BA 10), with a local maximum in the left hemisphere (Uchiyama et al., 2006; Wang et al., 2006a,b; Wakusawa et al., 2007; Shibata et al., 2010), and in the bilateral posterior cingulum, with a local maximum in the left hemisphere. In several studies, some of these areas were found active not only in irony, but also in ToM processing (Cavanna and Trimble, 2006; Abu-Akel and Shamay-Tsoory, 2011; Spreng and Mar, 2012), which is in line with the assumption of previous studies and a meta-analysis that ToM network is active while someone is understanding verbal irony (Bohrn et al., 2012; Spotorno et al., 2012; Obert et al., 2016; Van Ackeren et al., 2016).

During the ironic statement phase the SG as well as the RG activated regions associated mostly with linguistic and cognitive processing. The *SG* engaged the left IPL, which is well known as an area involved in language comprehension (Catani and Jones, 2005), and also in non-literal language processing (Rapp et al., 2012). In line with the findings of Rapp et al. (2012) we propose that in the present research the IPL activity might reflect a higher demand to integrate non-literal meaning into the context. Nevertheless, the local maxima of the left IPL activation lies within the region of the dorsal section of the temporo-parietal junction. In Kronbichler et al. (2017) this region was found to be over-activated in schizophrenia, suggesting cognitive or attentional processing as compensatory effects during ToM processing.

The SG also activated the pars triangularis of the left IFG. This region has been considered to play a semantic role in irony processing (Rapp et al., 2012; Obert et al., 2016). It is presumed that the left IFG is involved in the recognition of contradictory information between the context and the non-literal sentence meaning, and it works together with the right IFG, which is responsible for the detection of the incongruity between the statement and the prosody (Obert et al., 2016).

The *RG* showed activations in the posterior part of the left STG, in the right STS and in the pars triangularis of left IFG during understanding the ironic statement. BOLD responses in the left STG and in the left IFG were observed during semantic processing (Giora et al., 2000; Uchiyama et al., 2006; Wang et al., 2006b; Shamay-Tsoory and Aharon-Peretz, 2007), and during the semantic integration process in irony interpretation (Rapp et al., 2012; Obert et al., 2016). In addition, STS was also found to contribute to irony processing, explained as a higher-order linguistic processing (Shibata et al., 2010).

### Between-group activations during the ironic statement phase (irony vs. control task contrasts)

In the between-group comparison, during the ironic statement phase, the CG exhibited considerably greater BOLD responses in four brain regions compared to the SG: the right DLPFC, the right IPL, the left STG, as well as the left IPL. As it was described earlier, the right DLPFC has an important role in control mechanisms for context processing (Kompus et al., 2009) and in decision making in risky social situations (Rodrigo et al., 2014), while the right IPL plays a crucial role in self-other discrimination (Decety and Sommerville, 2003; Uddin et al., 2006).

In previous publications about semantic (Vigneau et al., 2006) and figurative language processing (Rapp et al., 2012; Obert et al., 2016) in healthy subjects, the activation of the left STG was observed. The superior temporal areas are involved in the analysis of verbal stimuli to extract social meaning (Redcay, 2008; Obert et al., 2016). The left STG together with the left IPL may contribute to semantic integration processes in irony interpretation (Rapp et al., 2012; Obert et al., 2016). Interestingly, the brain areas in which the SG showed weaker activations are involved either in self-other discrimination/representation, or in the integration of semantic and pragmatic language information. In conclusion, we presume that the observed weaker activations during the ironic statement phase together with those of higher activations during the context phase could lead to the detected comprehension impairment of irony in the SG. On the other hand, increased activations of the presented regions in the CG compared to the SG confirm the role of the executive (Spotorno et al., 2012; Bosco et al., 2017b) as well as the language system (Rapp et al., 2012) in ironic statement processing.

The evaluation of the between-group differences during ironic statement comprehension showed significantly stronger activations in the left DMPFC and in the right IFG (pars triangularis) in the CG compared to the RG. A large body of studies shows that the MPFC is one of the core component of ToM (Van Overwalle and Baetens, 2009), which activates during the mentalization process irrespective of the stimulus formats (Schurz et al., 2014). However, the MPFC is also activated in non-literal language processing (Rapp et al., 2012), especially in irony comprehension, and it is thought to be responsible for the suppression of competitive interpretations during comprehension processing (Papagno and Romero-Lauro, 2010).

Beside the under-activation of the MPFC, the RG also under-activated the right IFG, which has been previously found to be active in figurative language processing. As we described earlier, bilateral IFG activity has a role in detecting or resolving incongruity during irony processing (Obert et al., 2016). While we found that the SG activated the IFG only in the left hemisphere during the comprehension of the ironic statement phase, we also found that the RG had the same activation pattern. As the right IFG is suggested to be responsible for the attentive decoding and cognitive judgment of the emotional cues in prosody (Frühholz and Grandjean, 2013), this result suggests that schizophrenic patients and unaffected relatives may use less prosodic information during irony processing (Leitman et al., 2006). Since we did not detect any significant differences in the performance of the irony tasks between the RG and CG, we can only speculate that this between-group difference in the right IFG may be due to the fact that the CG and the RG used different strategies to comprehend the ironic remark.

We can suppose that despite the weaker activations of important brain regions associated with irony processing, making an extra executive effort in order to understand the social context could lead to the satisfactory performance in irony tasks. Nevertheless, we cannot rule out a possible use of a compensatory strategy, for example the “reality-based shortcut strategy,” which was described by Gyori et al. (2004). According to Gyori et al. (2004), using the “reality-based shortcut strategy” requires the ability to compare the contextual reality of the story with the literal meaning of the utterance. In the case of irony interpretation, the two representations contradict each other, so the literal meaning of the utterance is simply turned into the opposite meaning.

### Overall discussion

To summarize, our aim was to determine if impairments of irony comprehension in first-degree relatives of schizophrenic patients would fulfill the familiality criterion of an endophenotype. On the one hand, the fMRI results of the present study seem to support our hypothesis that altered comprehension of irony is an endophenotypic marker of schizophrenia, as we detected markedly different brain activation patterns in the RG compared to the CG. In the between group comparisons, the RG showed intermediate activation patterns with less over-activated regions than the SG during the context phase, and less under-activated brain areas than the SG during the statement phase. On the other hand, the behavioral results do not support that impaired irony comprehension is an intermediate phenotype of schizophrenia as the performance of the RG was as good as that of the CG, while the SG scored lower in the irony tasks than the RG or the CG.

Nevertheless, our results suggest compensatory activation of the DLPFC in the RG during the context phase, which is an important brain region for executive functioning, and it was shown to be involved in the recognition of ironic speech (Bosco et al., 2017b). In addition, our results may reflect a compensatory strategy, possibly relying on IQ-dependent problem solving and analogical reasoning (Gyori et al., 2004; Brüne and Bodenstein, 2005; Andreasen et al., 2008; Varga et al., 2014). Based on our present findings, the RG seems to be more effective in compensating the altered neural activity during irony comprehension, and the effectivity of the used compensatory mechanism may explain the missing performance impairment in irony tasks processing. After all, the altered neural activity during irony comprehension might reflect an endophenotypic characteristic of unaffected relatives at genetic risk.

### Limitations

Our study has several limitations. First of all, the sample size was rather low, so the replication of our findings on a bigger sample is needed, and we suggest cautious interpretation of our data.

We did not assess the potentially confounding neurocognitive (e.g., executive functions, working memory, attention, etc.), basic ToM (first- and second-order), or the linguistic abilities of the participants. Although it would have been interesting to integrate these dimensions into our study design, our aim was only to investigate the functional brain imaging correlates of irony processing in first-degree relatives of patients with schizophrenia compared to a group of schizophrenic patients as well as a control group.

Another important limitation is the use of antipsychotic medication in SG. All patients with schizophrenia were on maintenance antipsychotic medication. Antipsychotics influence brain activity, however they have little reliable effect on social cognition (Kucharska-Pietura and Mortimer, 2013), and some earlier study did not find any correlation between irony comprehension and the antipsychotic treatment (Mo et al., 2008).

## Conclusion

To summarize, the direct comparison of irony task performance of RG and CG failed to reach statistical significance. However, our results show that despite good task performance, first-degree relatives of schizophrenia patients have significant alterations in the neural circuits during irony processing. Interestingly, the observed alterations in brain functions that were found in the RG were somewhat similar to those found in the SG. Both groups showed higher activations during the social context phase and weaker activations during the ironic statement phase compared to the CG. One might propose that these functional brain alterations might be manifestations of the effect of genetic liability to altered figurative language processing in schizophrenia.

## Author contributions

RH study design, stimulus construction, writing article, manuscript revision. EV study design, stimulus construction, fMRI and behavioral data collection, fMRI and behavioral data analysis, writing article, manuscript revision. AH study design, fMRI and behavioral data collection, behavioral data analysis. EH behavioral data collection, behavioral data analysis. BT behavioral data collection, behavioral data analysis. HB behavioral data collection, behavioral data analysis. TT project idea, writing article, manuscript revision.

### Conflict of interest statement

The authors declare that the research was conducted in the absence of any commercial or financial relationships that could be construed as a potential conflict of interest.
